# Consensus statement of the Spanish Society of Internal Medicine and the Spanish Society of Medical Oncology on secondary thromboprophylaxis in patients with cancer

**DOI:** 10.1007/s12094-020-02477-6

**Published:** 2020-09-03

**Authors:** T. Quintanar, C. Font, E. Gallardo, R. Barba, B. Obispo, C. Díaz-Pedroche

**Affiliations:** 1 Department of Medical Oncology, Hospital General Universitario de Elche y Vega Baja, Elche, Alicante Spain; 2 Department of Internal Medicine, Hospital Clinic, Barcelona, Spain; 3 Department of Medical Oncology, Parc Taulí Hospital Universitari, Institut d’Investigació i Innovació Parc Taulí I3PT, Universitat Autònoma de Barcelona, Sabadell, Spain; 4 Department of Internal Medicine, Hospital Universitario Rey Juan Carlos, Móstoles, Madrid, Spain; 5 Department of Medical Oncology, Hospital Universitario Infanta Leonor, Madrid, Spain; 6 Department of Internal Medicine, Hospital Universitario Doce de Octubre, Madrid, Spain

**Keywords:** Anticoagulants, Haemorrhage, Low-molecular-weight heparin, Neoplasms, Vitamin K, Venous thrombosis

## Abstract

Up to 20% of cancer patients will develop some manifestation of venous thromboembolic disease (VTD) during their clinical course. VTD greatly impacts morbidity, mortality, quality of life and pharmaceutical expenditure. In addition, both thrombotic relapse and major haemorrhages derived from VTD treatment are more likely in oncological patients. To make the decision to establish secondary thromboprophylaxis as an indefinite treatment in these patients, it is important to review all the risk factors involved, whether related to the disease, the patient or the prior thrombotic event. The objectives of this consensus of the Spanish Society of Internal Medicine (*Sociedad Española de Medicina Interna*—SEMI) and the Spanish Society of Medical Oncology (*Sociedad Española de Oncología Médica*—SEOM) are to establish recommendations that help assess the risk of recurrence of VTD and haemorrhagic risk in patients with cancer, as well as to analyse the evidence that exists on the currently available drugs, which will allow the establishment of a protocol for shared decision-making with the informed patient.

## Introduction

The advances in antineoplastic treatment of most solid tumours have resulted in an increase in overall survival in patients with advanced disease [1]. Managing complications of cancer treatment, therefore, has value in improving survival and quality of life. Up to 20% of cancer patients will develop some manifestation of venous thromboembolic disease (VTD) during their clinical course, making it one of the main cardiovascular complications from the first year after diagnosis [2] with a major impact on morbidity, mortality, quality of life, and pharmaceutical expenditure. Both recurrence of and major haemorrhages from VTD treatment are more frequent in cancer patients [3]. This has led researchers to explore other anticoagulant treatment options in this scenario. Low-molecular-weight heparin (LMWH) has been established as the main therapeutic tool based on its greater efficacy observed against vitamin K antagonists (VKA) in the CLOT study and other studies during the first 3–6 months of treatment [4, 5].

In recent years, several clinical trials have evaluated the efficacy and safety of direct-acting oral anticoagulants (DOAC) in cancer patients, which has allowed an expansion of the therapeutic repertoire. In the clinical practice guidelines of the main scientific societies [6–8], DOAC is not recommended as a therapeutic option for patients with gastrointestinal or genitourinary tumours, as these tumours are associated with an increased haemorrhagic risk [6, 8]. In select patients, dicoumarinic agents may be a therapeutic option for long-term anticoagulant treatment, particularly for those who are not receiving chemotherapy or other agents that can interact with them pharmacologically, and also only providing that adequate control of the international normalized ratio (values of approximately 2–3) can be proven. However, there is little evidence for optimal therapy after 6 months of treatment, which requires individualized decision-making for each patient [9, 10].

The objectives of this consensus of the Spanish Society of Internal Medicine (*Sociedad Española de Medicina Interna*—SEMI) and the Spanish Society of Medical Oncology (*Sociedad Española de Oncología Médica*—SEOM) are to review the available evidence and establish recommendations, when possible, to help assess the risk of recurrence of VTD and the haemorrhagic risk in cancer patients. To do this, the rates of comorbidities, potential pharmacological interactions, and other factors must be taken into account to decide the best possible therapeutic management. Establishing a standardized evaluation protocol should facilitate shared decision-making with the informed patient and continuous re-evaluation according to the oncological evolution of the disease and the complications that have occurred.

## Factors influencing decision-making

### Factors related to the disease

Table 1 describes the considerations for and against the maintenance of long-term secondary thromboprophylaxis related to tumour pathology.

**Table 1:** Cancer factor considerations for and against maintaining long-term secondary thromboprophylaxis

For	Against
High thrombogenic risk:	Low thrombogenic risk:
Pancreatic cancer	Breast cancer
CNS tumours	Prostate cancer
Gastroesophageal cancer	
Histology of adenocarcinoma	Other histologies
Tumours with high degree of differentiation (G3)	
Locally advanced or metastatic disease	Localized disease
Presence of molecular alterations in *KRAS*, *ALK*, and *ROS*	Absence of molecular alterations
Recent surgery	
Hormone therapy in breast cancer	
Treatment with cyclin inhibitors in breast cancer	
Cisplatin chemotherapy treatment	
Antiangiogenic therapy	

*CNS* central nervous system

#### Location of the primary tumour and vascular compression

The presence of cancer increases the risk of VTD four to sevenfold with respect to the general population, such that approximately 20% of the episodes of thrombosis observed in the community will occur in cancer patients. Not all tumours have the same thrombogenic potential [11]. Pancreatic cancer, central nervous system tumours, and gastroesophageal cancer have the highest annual incidence of thrombosis (6–14%). At an intermediate level are renal cancer, lung cancer, ovarian cancer, and haematological tumours (3–4%), while cancers such as breast, prostate, and colon have a low thromboembolic risk (1–3%).

The aetiopathogenesis of VTD in cancer patients is complex and multifactorial. The state of hypercoagulability, endothelial damage caused by treatments, and circulatory stasis are contributory factors. Circulatory stasis may be caused by the limitation of the patient's mobility, as well as by the extrinsic vascular compression exerted by the tumour itself or its metastases (e.g., tumours with bulky lymphadenopathies).

#### Histology and stage of the disease

In some neoplasms, certain histological subtypes are associated with greater thrombotic risk, for example, in ovarian cancer and lung cancer, although this has not been seen in the different histological subtypes of breast and colon tumours. Patients with adenocarcinoma of the lung have a higher risk of thrombosis than those with squamous-cell carcinoma [12]. In addition, an increased risk of thrombosis has been observed in patients with mucin-producing adenocarcinomas such as those of the pancreas and gastrointestinal system.

The published results of the Vienna-CATS study have shown that the degree of tumour differentiation is associated with the risk of thrombosis: Patients with high-grade tumours (G3) have twice the risk of developing a thrombotic event than those with low-grade tumours (G1 or G2) [13].

The risk of thrombosis also increases with the tumour stage. Locally advanced and metastatic disease is associated with a higher risk of thrombosis than localized disease. The risk of VTD in patients with cancer without distant involvement is up to 4 times higher than in the general population and can increase to up to 58 times with respect to the general population if the patient has disseminated disease [14]. Patients with solid tumours and disseminated disease have 20 times the risk of those with tumour pathology but without metastasis. Finally, the risk of recurrence of thrombosis is higher in patients with advanced disease than in patients with localized disease (5 versus 2–3 times) and in patients with active disease.

#### Molecular alterations (KRAS, ALK, ROS1)

*KRAS* mutation is a predictive biomarker commonly used in colorectal cancer to identify patients who will not benefit from treatment with drugs against epidermal growth factor receptor (EGFR). Regarding the risk of thrombosis, there is evidence that the expression of tissue factors on the surface of colon tumour cells is activated by *KRAS* oncogene mutations and inactivation of the *TP53* tumour suppressor gene. Given that tissue factor is a procoagulant associated with systemic hypercoagulability, a multicentre study was designed to test whether patients with *KRAS* mutations had a higher risk of thrombosis. Results showed that patients with mutated *KRAS* colorectal cancer had 3 times the risk of developing thrombotic events as those with unmutated *KRAS*, making it the first study that showed the implication of a genetic mutation on the risk of developing thrombosis [15].

In 3–5% of patients with lung adenocarcinoma, *ALK* gene rearrangements are detected. There are now three drugs directed against this alteration (crizotinib, alectinib and ceritinib). In 2017, a study showed that patients with *ALK* rearrangements had a three to fivefold higher risk of thrombosis than patients with non-small-cell lung cancer (NSCLC) without *ALK* rearrangements [16].

In April 2019, an increased risk of thrombosis was reported in patients carrying *ROS1* translocation, similar to that seen in carriers of *ALK* rearrangements [17].

#### Treatment of the disease

##### Surgery

The risk of thrombosis is increased in all patients who undergo surgery, both due to the prothrombotic state associated with cancer and the high morbidity and complexity of surgery in these patients. In patients with cancer, the risks of deep vein thrombosis (DVT) and pulmonary thromboembolism (PTE) are up to 2 and 3 times higher, respectively, than they are in patients not requiring surgery.

Over the years, the risk of thrombosis associated with surgery has decreased due to the improvement of surgical techniques, the rapid mobilization of patients after surgery and the improvement of prophylaxis and perioperative care. Surgery performed on the pelvis and abdomen is associated with an increased risk of thrombosis.

##### Hormone therapy and cyclin inhibitors

Hormone therapy for breast cancer is associated with an increased risk of thrombosis, especially in patients treated with tamoxifen compared to those treated with aromatase inhibitors.

Recently, cyclin inhibitors (palbociclib, abemaciclib and ribociclib) have been incorporated into the treatment of metastatic breast cancer positive for hormone receptors and negative for human epidermal growth factor receptor 2 (HER2), and they are administered in combination with hormone therapy. Among their side effects, an increased risk of thrombosis (up to 5%) has been described, and there is debate about their pathophysiological mechanisms [18].

##### Chemotherapy

Cancer patients undergoing chemotherapy are 6–7 times more likely to develop a thrombotic event than those not receiving this treatment. Khorana et al. demonstrated that the thrombosis rate was higher in a cohort of cancer patients 12 months after starting chemotherapy (12.6%) than in the control cohort (1.4%) [19].

In addition, an increased risk of thrombosis has been associated with different types of chemotherapy. Cisplatin is one of the most widely used drugs for malignant tumours, usually in combination with other drugs, and published evidence links it with an increase in venous and arterial thrombosis [20]. The combination of cisplatin with other chemotherapies carries up to twice the risk of thrombotic complications in gastroesophageal tumours as with regimens with platinum salts, such as oxaliplatin (7% with cisplatin versus 1% with oxaliplatin). Carboplatin, another commonly used platinum salt, is considered to have an intermediate thrombotic risk (5.5 vs. 7.0% with cisplatin).

Other immunosuppressive and chemotherapeutic drugs, such as thalidomide, lenalidomide, gemcitabine, l-asparaginase, anthracyclines, fluoropyrimidines, and irinotecan, have also been associated with an increased risk of thrombosis [21].

##### Angiogenesis inhibitors

The pharmacological inhibition of the physiological functions of the vascular endothelial growth factor (VEGF)–VEGF receptor (VEGFR) axis affects the maintenance and protection of the normal endothelium, which may contribute to a greater predisposition to arterial and venous thromboembolic complications. However, the published data do not show a clear causal relationship between the use of antiangiogenic monoclonal antibodies (such as bevacizumab, aflibercept and ramucirumab) and the increased risk of VTD when drug administration is adjusted for duration by units of time. An increase in the risk of VTD was not observed with the use of oral antiangiogenic tyrosine kinase inhibitors. However, antiangiogenic therapy significantly increases the incidence and risk of arterial thrombosis [22]. In addition, elderly patients are at an increased risk of stroke.

### Factors related to the patient

VTD is a pathology of multiple causes, which include risk factors associated with hypercoagulability both acquired and genetic, and specific to each patient, that overlap the risk of thrombosis from cancer and its treatment. Thrombotic risk factors can also be categorized as transient (and potentially modifiable) or persistent. Likewise, thrombotic events themselves can be categorized as provoked or unprovoked, depending on the risk factors related to VTD [23–26]. The various factors that may have contributed to the development of the initial thrombotic event should be considered to estimate with greater precision the eventual risk of recurrence of thrombosis. Similarly, in the evaluation of the patient, personal characteristics that could increase the haemorrhagic risk during anticoagulant treatment should be considered [27, 28]. The balance between thrombotic and haemorrhagic risk factors allows clinicians to individualize the benefit-risk ratio associated with the decision whether to maintain the treatment of secondary thromboprophylaxis in each patient, and whether to adjust its intensity to a full or a medium- or long-term prophylactic anticoagulant dose (Table 2).

**Table 2:** Patient-related factors favouring recurrence of thrombosis and bleeding during anticoagulant treatment

Factors favouring the recurrence of thrombosis	Factors favouring haemorrhagic risk
Demographic factors	
Age < 65 years old Female sex African–American vs*.* Asian race Pregnancy and postpartum	Age > 65 years, in particular > 75 years
Lifestyle	
Sedentary Immobility Smoking	Immobility Reduced functionality Risk of falling High-risk professions and sports Alcohol abuse
Comorbidities	
Obesity Autoimmune diseases Inflammatory bowel Vasculitis (e.g., Behçet) Antiphospholipid syndrome Myeloproliferative syndromes Monoclonal gammopathy Nocturnal paroxysmal haemoglobinuria Anaemia or transfusions Thrombocytosis Leukocytosis	Previous bleeding, particularly if the cause is not corrected Severe renal insufficiency or uraemia Portal hypertension Hepatic failure Previous stroke Diabetes mellitus Anaemia Thrombopenia
Concomitant pharmacological treatments	
Erythropoietin Hormonal contraceptives Tamoxifen Corticotherapy	Antiplatelet agents Poor VKA-INR control
Genetic factors	
Congenital thrombophilia Deficiency of: Protein C Protein S Antithrombin Factor V Leiden Prothrombin G20210A Personal or family history of VTD	Haemophilia Von Willebrand disease Rendu–Osler–Weber disease
Mechanical factors	
Venous catheter Other endovascular devices Vascular compression Varicose veins, varicectomy Vena cava agenesis	

*VKA* vitamin K antagonist, *VTD* venous thromboembolic disease, *INR* international normalized ratio

Regarding demographic characteristics, younger age (< 65 years) has been associated with an increased risk of recurrence in cancer patients [3, 29], while advanced age (≥ 65 years) carries both a thrombotic and a haemorrhagic risk in the general population on anticoagulants for VTD [27, 28]. There are other, more common haemorrhagic risk variables, although these are not exclusive to the geriatric population, such as the risk of falls, immobility, renal failure, and the concomitant use of antiplatelet therapy due to the previous cardiovascular disease. Female sex has been associated with an increased risk of recurrence of thrombosis in patients with cancer [30], and it is a variable included in the Ottawa thrombosis recurrence predictive model along with other oncological variables and previous thromboembolic episodes [31]. However, the Ottawa model has not been validated consistently in other cohorts of patients, and its practical utility is limited [31]. In fact, among patients with "unprovoked" thrombosis, the risk of VTD is higher in men. Some data suggest that there is a greater risk of thrombosis in African-American cancer patients than those of Asian origin [32, 33], although there are no data on the risk of recurrence or long-term bleeding according to race or ethnicity [9, 34]. Both sedentary lifestyle and smoking are associated with increased thrombotic risk, while alcohol consumption can increase the risk of haemorrhagic complications. In an analysis of the RIETE registry, immobility was also independently associated with increased haemorrhagic risk [29]. In this sense, it is of interest to take into account other aspects of profession and lifestyle, such as participating in sports, that may help promote healthy lifestyle habits and provide an opportunity to improve thrombotic or haemorrhagic risk.

Regarding the presence of comorbidities, the coexistence of infection, arterial thromboembolism, kidney disease, lung disease, trauma, or arthrodegenerative, neurological or psychiatric diseases that lead to immobility can increase the risk of thrombosis. Similarly, myeloproliferative syndromes, nocturnal paroxysmal haemoglobinuria and haematological diseases that present with anaemia, leukocytosis, or thrombocytosis can also increase the risk (Table 2). Among autoimmune inflammatory diseases that increase the risk of thrombosis are inflammatory bowel disease, antiphospholipid syndrome and vasculitis with endothelial involvement (Behçet disease). The presence of previous venous disease, or a central venous catheter or other intravascular devices also increase the risk, along with other mechanical factors that can increase venous stasis, such as vascular malformations or obesity.

Comorbidities that increase the haemorrhagic risk include a history of recent major haemorrhage and diseases associated with digestive bleeding (peptic ulcer, intestinal angiodysplasia or colonic polyposis) or bleeding in other locations, particularly if the cause is not corrected. Renal and hepatic insufficiency, portal hypertension and the presence of anaemia and platelet disease are also associated with increased haemorrhagic risk.

In the presence of such comorbidities, the use of other medications that may reduce thrombotic risk should always be considered, such as erythropoiesis-stimulating agents, thalidomide, and hormonal therapy in young premenopausal patients for contraceptive purposes or as adjuvant treatments in survivors of hormone-sensitive neoplasms. In contrast, antiplatelet drugs increase the haemorrhagic risk. In general, the possibility of pharmacological interactions that may increase or decrease the effect of anticoagulant therapy should be carefully reviewed, particularly if long-term use of VKA or DOAC is planned.

Finally, the possibility of genetic thrombotic risk factors or congenital thrombophilia should be taken into account, such as deficiency of natural anticoagulants (very infrequent with an estimated prevalence < 1% in the general population) including antithrombin, protein C, and protein S. The presence of factor V Leiden and prothrombin G20210A (PTG20210A) is more prevalent in the general population, although their screening in routine clinical practice is not recommended in most patients with VTD. If such genetic factors are present, they must be interpreted individually as to whether they are thrombotic risk factors. In any case, the personal and family history of first-degree VTD should be taken into account in any decision-making [35, 36].

### Factors related to prior thrombotic events

To decide whether anticoagulation should be indefinite, the thrombotic event must be evaluated taking into consideration its location, severity, and sequelae (Table 3).

**Table 3:** Factors related to thrombotic events for and against initiating secondary thromboprophylaxis

For indefinite treatment	For withdrawal
Thrombotic event	
Pulmonary embolism Proximal vein thrombosis Venous thrombosis in upper extremities with catheter maintenance Acute unprovoked symptomatic multivessel splenic thrombosis CNS thrombosis	Distal deep vein thrombosis Venous thrombosis in upper extremities with catheter removal Isolated, chronic, or postsurgical splenic thrombosis
Symptoms and/or tumour burden	
No influence VTD without predisposing factors	Subsegmental asymptomatic incidental pulmonary embolism (false positive) Post-surgical VTD
Aftermath	
Chronic VTD with or without pulmonary hypertension. In patients with persistent dyspnoea, high central tumour burden, high-intermediate-risk PTE, high-risk PTE, or acute pulmonary hypertension. Evaluate lung scintigraphy or echocardiogram at 3–6 months	Absence of residual thrombosis

*VTD* venous thromboembolic disease; *CNS* central nervous system; *PTE* pulmonary thromboembolism

#### Location of the prior thrombotic event

The location of an initial thrombotic event influences the decision about duration of anticoagulant treatment. In patients with pulmonary embolism, mortality from the recurrence of the thrombotic event is higher than in those with DVT [37, 38], and therefore, in these patients, it is particularly advisable to maintain anticoagulation if the cancer is active. Along the same lines, proximal venous thrombosis of the lower extremities is associated with an increased risk of pulmonary embolism, and the duration of treatment should be longer than in distal or upper-extremity thrombosis, in which the risk of pulmonary embolism is lower; therefore, a shorter duration of treatment should be considered. However, distal thrombosis in patients with cancer followed in the long term has a worse prognosis than in patients without cancer, with a higher haemorrhagic risk and rethrombosis risk, and so treatment decisions should be individualized [39].

Thrombosis of the splanchnic territory is increasingly common in cancer patients, although it is not suspected in up to 59% of cases [40]. Although patients with cancer and splanchnic thrombosis have more comorbidities that increase the risk of haemorrhage (e.g., platelet disease, impaired liver function, gastrointestinal disease or gastrointestinal cancer), in more than 50% of patients treatment is prolonged beyond 6 months with LMWH doses similar to those administered to patients with thrombosis in other areas of the body, with the first month into anticoagulation presenting the highest risk of bleeding [40].

There are no clinically significant differences in the risk of major haemorrhage or recurrence depending on whether the thromboembolic episode is incidental or symptomatic [40, 41], and therefore, it should not determine the duration of treatment. However, the number of affected vessels (vascular territory at risk), the chronicity of the lesions, their relationship with surgery and the haemorrhagic risks are the main factors that must be taken into account to prolong anticoagulation. In distal or upper-extremity thrombosis in cancer patients, their relationship with the catheter guides decisions. A total of 3–4% of patients with cancer and permanent central venous catheters will develop catheter-related thrombosis within one year [42]. The guidelines do not recommend removing the central catheter unless it is faulty, contaminated and/or the clinical symptoms worsen despite anticoagulation [7, 43]. In addition, it is advisable to maintain anticoagulation, while the catheter is in place. If the catheter is removed, the risk of recurrence of thrombosis is low, and therefore, it is not necessary to maintain anticoagulation [44]. Finally, in venous thrombosis of the central nervous system, indefinite treatment is usually recommended unless the initial episode is related to the administration of drugs (e.g., chemotherapy and/or hormonal treatment) that can be suspended [45].

#### The severity of the prior thrombotic event

The mortality rate, the relapse rate, and the risk of major haemorrhage in patients with cancer are similar to those of non-oncological patients with symptomatic pulmonary embolism or incidental pulmonary embolism [46]. However, in patients with untreated incidental pulmonary embolism, the risks of recurrence and death are higher than in anticoagulated patients, without significant differences between patients with central and subsegmental embolism [47]. Therefore, in most guidelines, anticoagulation is recommended in patients with cancer and isolated incidental subsegmental pulmonary embolism even if it is asymptomatic, but whether the diagnosis is correct should be evaluated by having images reviewed by an expert radiologist and a complete study carried out using venous Doppler ultrasound of the lower extremities [48].

#### Sequelae of the prior thrombotic episode

In patients with cancer, the sequelae of a prior thrombotic event should also be evaluated. Thus, chronic thromboembolic pulmonary hypertension is an indication of indefinite anticoagulation, and cancer is a risk factor for its development. The role of residual thrombosis (RT) in the lower extremities in the prediction of recurrence is not clear. In patients with cancer, the absence of thrombi in the re-evaluation of the episode at 6 months means that recurrence is unlikely [49], and therefore, the decision to withdraw anticoagulation is reinforced in patients with medium–high haemorrhagic risk, but the presence of RT does not increase the risk of recurrence.

## The contribution of previous diagnostic tests to decision-making

The use of RT as a predictor of recurrence after DVT has not been validated due to the use of different diagnostic techniques and testing times and the absence of a standard definition. In a meta-analysis of 2527 patients with DVT without cancer, the predictive power of RT was weak (hazard ratio [HR]: 1.32; 95% confidence interval [CI 1.06–1.65) and absent when ultrasound was performed beyond 3 months (HR 1.19, 95% CI 0.87–1.61). RT was observed in 55% of patients, and relapse was observed in 16% [50]. In a study of 347 patients with cancer and DVT, 70% of whom had RT, participants were randomized to maintain or not maintain anticoagulation for more than 6 months, but no significant differences were observed in relapses (HR 1.37, 95% CI 0.7–2.5; *p* = 0.311) [49]. The absence of RT was a powerful predictor of nonrecurrence of DVT, as there was a 3% recurrence rate after stopping anticoagulation (HR 6.0, 95% CI 1.7–21.2, *p* = 0.005). However, the possibility of bias in that study means that the results remain inconclusive. In 153 patients evaluated (46% RT) in a post hoc analysis of a cohort study in patients with cancer and DVT, RT showed no predictive power of relapse (odds ratio [OR]: 2.30; 95% CI 0.73–7.22) [51].

D-dimer level has been correlated with the risk of recurrence in patients with DVT; therefore, a low D-dimer might suggest anticoagulation should be stopped, although there are no conclusive data to support this practice [52]. In cancer patients, D-dimer may be elevated, even without DVT. In a study of 167 patients with active cancer and a 12% incidence of VTD, D-dimer gradually increased in VTD patients and remained constant in patients without VTD and an increased risk of recurrence in case of duplication (HR 2.78; 95% CI 1.69–4.58; *p* < 0.0001) [53]. In another study with 114 patients with cancer and VTD and an incidence of relapse of 9%, the elevation of D-dimer and high-sensitivity C-reactive protein was predictors of relapse of the thromboembolic episode (D-dimer: HR 5.81; 95% CI 1.1–31.7; C-reactive protein: HR 9.82; 95% CI 19–52), and therefore, they may be useful to identify susceptible patients following the interruption of anticoagulation [54].

Therefore, given the limited evidence for parameters such as RT and D-dimer in prediction of VTD in cancer patients, it is not possible at this time to recommend an anticoagulation strategy.

## Options for secondary thromboprophylaxis

Here, the evidence for each of the therapeutic options is reviewed.

### Vitamin K antagonists

Few clinical trials have compared the efficacy of VKA versus LMWH in the long-term treatment of patients with cancer and VTD. In addition, most have a low or moderate degree of evidence, and patient follow-up is usually no longer than 12 months. In a Cochrane review published in 2018 [55], it was concluded that during the first year, the risk of recurrence was higher in patients treated with VKA than in those treated with LMWH (HR 0.58; 95% CI 0.43–0.77). Regarding safety variables, the differences in the risk of major or minor haemorrhage did not reach statistical significance, as was also the case with all-cause mortality. None of the studies included quality-of-life variables. Therefore, in terms of efficacy, the review concluded that VKA is less effective than LMWH in the long-term treatment of VTD, although they are similar in terms of safety. However, a recent study based on the RIETE registry has shown that patients treated with LMWH who switch to VKA after 6 months of treatment have a recurrence or bleeding rate similar to that of patients who continue with LMWH, so this treatment should be considered, especially in patients in which LMWH or DOAC are contraindicated (e.g., patients with creatinine clearance below 30 ml/min) [56].

### LMWH at full doses

LMWH is the treatment of choice during the first 6 months following a thromboembolic event. Beyond this period, evidence is scarce. The DALTECAN trial demonstrated, in 100 patients with cancer and VTD with a follow-up longer than 6 months [57], that dalteparin maintains its efficacy over time in terms of decreasing recurrence, with a stabilization of the haemorrhage rate, which was higher in the first month (3.6%) but was gradually reduced (0.7% in the seventh month). Similar results have been obtained with tinzaparin in treatment up to 12 months [58].

### LMWH at prophylactic doses

There are no studies to support the use of reduced doses of LMWH in cancer patients in whom long-term treatment is maintained [59]. It is recommended to administer the dose according to the actual, rather than the ideal, patient weight.

### DOAC

Two clinical trials have been reported in patients with neoplasia and VTD with a follow-up beyond 12 months. In the HOKUSAI trial, the noninferiority of edoxaban compared to LMWH was demonstrated when the combined objective of recurrence and haemorrhage was evaluated [60]. There were no differences in the relapse rate or mortality rate, but the incidence of bleeding was higher in patients treated with DOAC. In a post hoc analysis, the greatest haemorrhagic risk was observed in patients with gastrointestinal or urological neoplasms. In the SELECT-D trial [61], patients who received rivaroxaban had a lower recurrence rate and a clinically relevant higher haemorrhage rate than those treated with dalteparin, especially among patients with gastrointestinal or urological tumours, although there were no differences in mortality rate between treatment groups.

In addition to these studies, several meta-analyses have been performed, and the results show that although the risk of recurrence is reduced, the risk of bleeding is slightly higher [62]. It is possible that a higher net benefit is obtained in patients treated with DOAC if those with a low risk of haemorrhage are selected.

In the ADAM-VTD study [63], the risk of haemorrhage was similar in patients treated with apixaban or dalteparin, suggesting that this drug may be a good alternative. In the recently published CARAVAGGIO study, patients with active cancer and DVT or PE were randomized to receive apixaban or dalteparin for 6 months. The rate of rethrombosis was 5.6% in the group treated with apixaban compared to 7.9% in the group treated with dalteparin (HR 0.63; *p* < 0.001 for non-inferiority), with no increased risk of gastrointestinal haemorrhage in the first 6 months (1.9% with apixaban vs. 1.7% with dalteparin; HR 1.05) [64]. There are no results on the use of apixaban beyond 6 months, but one ongoing study is comparing a full dose (5 mg/12 h) with lower doses, simulating other studies in patients without cancer [65].

In summary, most of the guidelines consider treatment with DOAC, specifically treatment with rivaroxaban and edoxaban, to be a reasonable alternative to HPBM except in patients with gastrointestinal or urological tumours or with mucosal involvement and high haemorrhagic risk [66–68]. It is important to discuss with patients the different therapeutic options available, recognizing that for many, oral treatment is considered an advantage only if it is more effective than subcutaneous treatment [69].

### Antiplatelet agents

Currently, there is no evidence that antiplatelet agents can be useful in long-term secondary thromboprophylaxis in patients with cancer and prior VTD. Studies that demonstrate its efficacy have been performed in patients with unprovoked VTD, but excluding those with neoplasia [70].

## Shared decision-making with the informed patient

**Fig. 1:**
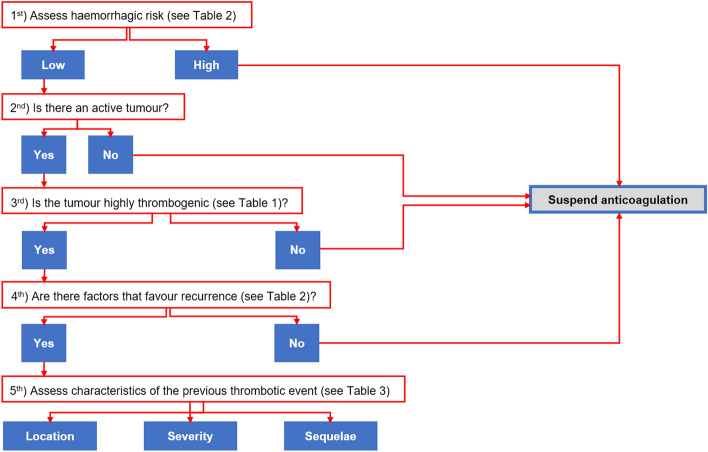
Considerations after 6 months

Precision or personalized medicine undoubtedly requires a proactive role for the patient in shared decision-making (SDM) [71, 72]. Assessing the benefits and drawbacks of each therapeutic option with the patient should complement the individualization of treatment and therapeutic adjustments based on the increasing availability of biomarkers and the predictive models of response or toxicity (Fig. 1). The role of the patient in SDM is particularly important in situations where the available evidence is limited and when discussing treatments that can have a high impact on the quality of life or the lifestyle of the patient [73, 74].

The patient must be able to participate in SDM, taking into consideration the advantages and disadvantages from several viewpoints [75, 76]: (1) whether or not to continue anticoagulationt; (2) establishing the expected duration of anticoagulation and the timelines to review and adjust if necessary; (3) assessing the possibility of correcting potentially modifiable risk factors for thrombosis; (4) considering accessibility and costs; and (5) considering the personal physical and psychological impact of each therapeutic option, compared with therapeutic abstention [74].

Despite the importance of SDM, its application to clinical practice is limited for several reasons. For example, tools for SDM have not yet been developed or validated in the field of cancer and VTD patients. Other limiting factors are the high workload, lack of time for consultations and difficulties in increasing education and awareness about the importance of thrombosis and anticoagulation in oncology. Undoubtedly, the research and development ongoing in these areas will help to integrate the available tools in a practical way in highly complex scenarios for oncological patients for whom oncospecific and supportive treatments coexist.

## Conclusions

VTD is one of the main cardiovascular complications of cancer patients, and therefore, it is of great importance to ensure its correct diagnosis and management to minimize its impact on morbidity and mortality. The improvements in oncological treatments in the last decade has increased the survival of cancer patients, and therefore, it is of vital importance to periodically review the evidence for the situation and treatment of thrombotic events, not only in the acute phase but also in the long term, especially where the scientific evidence has been weak. In reaching the decision on whether or not to continue anticoagulation beyond 6 months, it is important to review all risk factors involved (i.e., those related to the disease, the patient and any prior thrombotic event) and to establish a risk/benefit balance for that decision-making. If the decision is reached to continue with anticoagulant treatment, then the choice of the particular therapy to use must be made, which includes VKA, LMWH, or most recently DOAC, taking all factors and considerations into account and ensuring the patient’s full and informed participation in the decision-making process.
